# Abnormal Mucous Membrane

**Published:** 1874-05

**Authors:** S. B. Tizzard


### DENTAL REGISTER.

VOL. XXVIII.] MAY, 1874. [No. 5.

### ABNORMAL MUCOUS MEMBRANE.

BY DR. S. B. TIZZARD.

The diseases affecting the sott tissues of the oral cavity are of a character of great importance to us as dentists in the practice of the profession; for on their well being depends in a great measure the success of most of our operations.

The mucous membrane of the mouth seems to be more subject to the action of morbid influences in early life than during any other period, in consequence, possibly, of the many and rapid changes constantly taking place in the tissues at that time; because of the eruption of the temporary teeth and also in consequence of the greater susceptibility of the constitution to general organic disease. It is not confined to early childhood alone, or during the period of teething; but it affects children of all ages, taking different forms and producing different results in one case from another, owing to the health, habit and circumstances by which the child may be surrounded at the period of the attack.

The affection of the mucous membrane of the mouth most common at this time of life is termed stomatitis or inflammation. It is indicated in some cases, first, by simply an increased redness of the surface of the membrane unattended by any swelling; in other cases, it affects not only the mucous membrane but the adjacent parts, as, for instance, the submaxillary, sublingual and also the glands of the neck, causing extreme swelling and pain, though not of an intense character, unless deep seated in the tissues. In certain stages of the general health and constitution, there is a very great tendency to an ulcerative condition at times, attended with very serious consequences. During this condition, the saliva sometimes flows copiously; at others, the mouth seems dry and clammy, attended by a coated or furred tongue, through which covering little inflamed papillæ may be seen projecting. There is also a loss or vitiated condition of the taste, attended by difficulty during mastication and deglutition.

Simple, or erythematic stomatitis is very general at an early age, sometimes affecting the tongue alone, and, again, by reddened patches slightly raised and separated from each other, on the membrane, but gradually uniting, finally covering the whole cavity of the mouth.

It may be caused by gastric irritation, also by stimulating food; the most frequent constitutional causes are febril diseases. In treating stomatitis the remedies usually employed are very simple, consisting of mild laxative medicines, as vichy salts, magnesia, etc., and soothing mouth-wash of borax, honey or tolu. If the patient be a babe under treatment, being raised by hand, Dr. Tomes recommends the addition of a little lime water to the milk given it.

Another disease of this membrane common to infants is called thrush or apthæ, consisting of small white or grayish colored ulcers situated on the tongue, inside of the mouth and frequently extending to the throat and fauces.

It is caused by lack of food containing proper nourishment and by irritation of the bowels. It is usually attended with diarrhœa, though at first the bowels may be costive. It is seldom dangerous and in treating it the disordered state of the bowels must be removed by using ant. acid and astringent medicines, the general system being toned up by the proper administration of a tonic, as cod-liver oil or iron, with mouth-wash consisting of a dilute solution of borax, applying at the same time crystal carbolic acid to the ulcers themselves.

Ulcerative stomatitis, a disease to which our attention is often called, occurs in the anterior part of the mouth, except in very rare instances, and most frequently it commences at the labial margins of the gums of the inferior incisor teeth. They first become congested, inflamed and swollen, bleeding at the slightest touch. Unless attended at once, the gums rapidly become detached from the teeth, exposing their necks and also laying bare the alveolar processes beneath. This is frequently the case with children of poor people living in large cities in poorly-ventilated tenement houses, where, in consequence of neglect or ignorance, attention has not been called to the disease until it has become deep seated; in such cases the affection will not only cause the loss of the teeth, but even the alveolar process itself may become diseased and act as an irritating agent in aggravating the disease. The cheek or lip also, in contact with the affected part when the ulcers are extensive and the blood impure, become subject to very near the same condition, probably in consequence of some of the fungi becoming attached or transplanted from the ulcers to the membrane, which, lacking vitality in consequence of this impoverished condition of the blood to resist the destructive influence, readily falls beneath the acrid irritating action of this agent of disorganization

The exact causes are not wholly either of a local or a constitutional character; it may result from a disordered condition of the alimentary canal, and, again, in persons subject to diseases of this character, it may develope itself from any slight irritation of the tissues of the mouth.

In its treatment, should there be any constitutional trouble, proper remedies must be given for its removal. In cases of this nature, local applications alone can do but little good, except to slightly ameliorate the symptoms until the irritating cause be removed. For this purpose, rinsing the mouth with a solution of alum, lime water and borax, also chloride of zinc applied by penciling the part, will be found beneficial, as will also the presentation of chlorate of potash; for an infant one year old, 5 grains ought to be given every 4 to 6 hours, owing to severity of symptoms; for an adult, a tea-spoonful three times a day; for a child 3 or 4 years old, about three grains, taken in sugar; for a child from that age to ten, about a full dose, or 10 grains every three hours. If the bowels are at all costive, they should be moved by some gentle aperient. I have found vichy salts, as stated before, to be most excellent; for a child 4 or 5 years old, a half tea-spoonful of the salt in a table-spoon of sweetened water; for an adult, two tea-spoonfuls in a small half tumbler of water prepared as above mentioned. In case the patient should be of a syphilitic diathesis, so constitutionally predisposed to this trouble, the administration of the iodide of potash—if an infant, $\frac{1}{2}$ gr.; if a child from 2 to 4, 2 gr.; if an adult, 5 gr.—once in 6 hours will be found of very great benefit. Crystal carbolic acid applied to the ulcers will be found very beneficial in promoting healthy granulations. In case the ulceration has extended to the cheek, it will be best to place a small pledget of cotton between the gum and cheek, with a dressing of dilute carbolic acid and glycerine to prevent adhesion. Should the trouble be of a strictly local character, it may readily be relieved by the removal of the irritant, whatever that may be, followed by a detergent and stimulant wash.

Another disease to which this membrane is liable among infants, but one that happily is of rare occurrence, is termed gangrenous stomatitis. It is met with among children from 2 to 8, but most commonly in infants from 2 to 4 that have been surrounded by about the same conditions as were described among the exciting causes of ulcerative stomatitis. Children predisposed to it are of the lymphatic temperament; debilitated constitutions; pale, flabby skin; and weak digestive organs where the functions of nutrition are but imperfectly performed.

Among the first symptoms usually noticed, predictive of this disease, is an inflamed condition of the gums, followed by general helplessness, peevishness, loss of sleep and appetite, with inordinate thirst; the skin becomes pale, countenance sad, and there is a singular puckering about the corners of the mouth. These symptoms last but a few days, and again for weeks, before the disease becomes fully developed; when this is the case, the face begins to swell rapidly, the skin shining, glossy and very hard, painless to the touch and having in its center a splotchy-looking red spot.

Much, we might say everything, depends upon the time the treatment is begun, if a cure is effected. The one great reason why this disease is so frightfully fatal, is the fact that, being, as it is, almost wholly devoid of attendant pain, it attains such an intensely malignant character before being noticed, that it is impossible to eradicate its destructive influence.

The treatment of this frightful malady, if gangrene has actually begun, consists in the destruction or eradication of the diseased portion by the use of strong caustics, such as nitrate of silver, fumes of nitric acid; followed locally by a detergent and cleansing mouth-wash: as, chlo. zn. solution, 10 gr. to pint of water; liquor chlorinaten soda, Labaraque solution, 1 ʒ to 3 ℥ of water; also a solution of permanganate of potash, 1 to 6 ʒ to pint of water: and in administering internally milk, strong beef-tea, cod liver, and also by tonics of iron to strengthen the general system.

However, much more may be done towards preventing the occurrence of the disease, by change from an impure to a pure atmosphere; good, healthy diet; cleanliness and by proper attention given to toning up the system, than curing the disease after it has once become fully developed.

Among other diseases to which this membrane is subject, is that termed mercurial stomatitis, or active inflammation. Usually the first indication of the presence of this disease is a peculiarly disagreeable fetor of the breath. followed by a metallic or coppery taste, with an increased flow of saliva. The margins of the gums of the inferior incisor teeth first become inflamed and slightly swollen; the redness rapidly extends over the whole membrane until the mouth is more or less diseased, owing to the amount of the drng presented.

These symptoms are followed by an uneasy feeling about the gums a little above the free margins of which a white line may be seen; the teeth become sensitive to pressure, often aching; the saliva flowing incessantly; the muscles of the jaw stiff and painful, frequently to such an extent that it is impossible to open the mouth, masticate and swallow; arrived at this stage, the odor of the breath, before bad, becomes intolerable; ulceration sets in, commencing around the necks of the teeth, which in consequence become loosened, also affecting the lips, cheeks and fauces. Severe cases are frequently attended by profuse hemorrhage, sloughing of the tissues, also by fever, sometimes symptomatic, and again induced by the administration of mercury. Fatal results have occasionally occurred in consequence of the debilitating effects of irritation on the constitution, hemorrhage and lack of proper sustenance; though persons usually recover after very severe attacks, though not without more or less deformity of the mouth.

In ordinary salivation, the most that will be necessary to do will be to discontinue the use of the mercury. In case the gums still continue swollen, soft and spongy, a milk diet should be prescribed, a mild laxative given, followed by the presentation of potassa chloras of 8 to 10 gr. in ℥ss of water —tea-spoonful 3 or 4 times a day; same also used as a soothing mouth-wash, of the strength of a tea-spoon to an ounce of water. It will be found very useful in allaying the inflammation in case the chlo. potash should not act readily. Iodide of potash, given in doses of 3 to five gr. three times a day, will be found beneficial. If any of the teeth should have become loosened, and astringent wash will be indicated; bleeding the gums will also be found useful in assisting to restore them to health. In removing the fetor of the breath a wash of phenol sodique, or chlorinated soda, in proportion of 1 to 2 tea-spoonfuls to a tumbler of water, will be found excellent.

The last morbid condition of this tissue and the gums to which we shall call your attention is that of chronic inflammation—a disease rarely attacking persons, so that it may be noticed, before arriving at the age of 34 or 35, or until middle life. It is very insidious in its encroachments on the tissues of the mouth and is usually the most difficult to cure of any of the the diseases to which our attention may be called. In its first advances there will be noticed a slightly blunted and thickened condition of the free margin of the gum, attended by a soft, white, cheesy exhalation, seemingly thrown from off the the tissue; also a lack of that greatly increased redness which usually characterizes inflammation of this tissue, except in occasional cases, where it may range from a light pink to a purple color. The membrane covering the surface of the gum, instead of being smooth, presents the appearance of being drawn over a surface on which are strewn little round balls of some substance, giving it a peculiar roughness that when once seen is always easily recognized as a symptom of the occurrence of this disease. It is apt, when of a constitutional nature, to affect all the teeth alike; though cases of this character are of somewhat rare occurrence. In its usual form it attacks but two or three teeth, often but one. The teeth that in my practice it has seemed most prone to attack have been the superior incisors and also the first molar teeth of the upper jaw. This may seem somewhat strange, as our attention is so frequently called to the fact that the lower incisors, when neglected, so often become loose; but I have found in almost, if not in every case, that this may be directly traced to the pernicious influence caused by the deposition of salivary calculus. When but one tooth (usually an incisor) is affected, the disease may be termed local, and is seldom attended by any pain or inconvenience, except that of increasd length, which, from its exposed situation, makes it very apparent and, to many, a source of great annoyance on that account. In the more advanced stage of the disease, where several teeth, regardless of their being perfect or decayed, are attacked, there is a slight discharge of yellow, purulent matter from between the gums and necks of the teeth, very apparent on pressure. This condition is sometimes unattended by pain; again, the teeth are sensitive to the pressure of the teeth during mastication, and the jaws or part affected becomes the seat of great continued, or *again*, of intermittent pain, at times neuralgic in its nature. If the disease is allowed to pursue its course unchecked, the matter becomes very offensive and profuse, the breath fetid; not only the teeth, but the alveolus itself may become diseased, attended by extensive absorption; following which, the teeth become so loose as to drop out in consequence of their complete disconnection from the dental periosteum by which they were enveloped. It frequently happens that, in consequence of the removal of periosteum from the body of the root, that small particles of tartar may be deposited on the exposed surface, which in turn excites no little irritation, and no doubt aggravates, though it is not the cause of, the disease. In some persons, where there is a predisposition to periosteal inflammation, it may be caused locally by the presence of any irritant, no matter how slight; such as the sharp edge of an old root or fang, by the rough edge of a filling or by any other agent that will excite and by its presence keep up a morbid irritation. Again, among the constitutional causes may be mentioned mercurial poisoning, chronic dyspepsia or any systematic disease which may increase the liability of the mucous membrane and gums to morbid influence.

In the treatment, a proper diagnosis as to whether it is of a constitutional or local character must first be made; all local irritants be thoroughly removed, such as tartar, decayed or badly diseased teeth, roots or any agent whatever that may be productive of irritation. If the gums be congested, free lancing should be resorted to, followed by some tonic astringent mouth-wash, such as krameria, full strength. I have also found topical applications of iodine, tr. of capsicum, beneficial; and also, after thoroughly cleansing the root or part affected, the most efficient remedy that I have yet used has been the deliquescent salt chloride of zinc, by passing a broach around which was wrapped a few fibers of cotton, saturated, up underneath the loosened portion as far as it would go, and passing completely around the tooth. I have also used a pointed piece of wood for this purpose, and found it to work well. In removing the fetor carbolic acid will be very useful, as will also phenol sodique and liquor chlorinated soda, applied in the same manner. To correct the breath, prepare the two last named as for use in mercurial stomatitis.

If syphilitic in character, 5 gr. of iodide of potash may be given three times daily, using at the same time local treatment; if it should be caused by dyspepsia, proper attention should be given to restoring the stomach to health by tonics, exercise and proper diet.

